# Cardiovascular Assessment Before Kidney Transplantation: Bridging the Gap Between Guidelines and Clinical Practice

**DOI:** 10.3390/jcm15218565

**Published:** 2026-11-03

**Authors:** Andrea Barbieri, Giacomo Mori, Filippo Bondi, Alessio Bragagni, Alice Parmigiani, Niccolò Morisi, Floriana Cannito, Gaetano Alfano, Gian Piero Guerrini, Barbara Catellani, Giuseppe Boriani, Gabriele Donati

**Affiliations:** 1 Cardiology Division, Department of Biomedical, Metabolic and Neural Sciences, University of Modena and Reggio Emilia, Policlinico Di Modena, 41124 Modena, Italy; 2 Nephrology, Dialysis and Kidney Transplant Unit, Azienda Ospedaliero-Universitaria di Modena, 41124 Modena, Italyniccolo.morisi@unimore.it (N.M.); gabriele.donati@unimore.it (G.D.); 3 Anesthesia and Intensive Care Unit, University Hospital of Modena, University of Modena and Reggio Emilia, 41124 Modena, Italy; 4 Surgical Medical and Dental Department of Morphological Sciences, University of Modena and Reggio Emilia, 41124 Modena, Italy; 5 Hepato-Pancreato-Biliary Surgery and Liver Transplantation Unit, University Hospital of Modena “Policlinico”, University of Modena and Reggio Emilia, 41124 Modena, Italy

**Keywords:** kidney transplantation, cardiovascular assessment, coronary artery disease, risk stratification, stress echocardiography, exercise capacity, physiological vulnerability, wait-list surveillance

## Abstract

Cardiovascular assessment before kidney transplantation (KT) remains uncertain despite contemporary guidelines. Historically, many transplant centers have adopted intensive screening strategies to detect obstructive coronary artery disease (CAD), often resulting in stress testing, coronary angiography, revascularization, dual antiplatelet therapy, and potentially delayed transplantation. However, evidence that systematic detection and prophylactic treatment of stable CAD improve outcomes in asymptomatic KT candidates remains limited. Recent American, British, and European guidance increasingly supports more selective, risk-adapted assessment, yet the low level of supporting evidence leaves substantial discretion in patient and test selection, as well as wait-list surveillance. This implementation gap is particularly relevant for historically high-testing centers seeking to revise established protocols. Moreover, the cardiovascular phenotype of advanced chronic kidney disease extends beyond obstructive epicardial CAD and includes myocardial, microvascular, structural, and systemic abnormalities that may be better captured by physiological vulnerability and functional reserve. Exercise-based assessment may therefore provide prognostic information beyond ischemia detection. In this Viewpoint, we examine the evidence challenging intensive CAD-centered screening, discuss the evolving role of functional testing and risk-adapted surveillance, and illustrate how these principles were translated into a multidisciplinary local protocol revision. The emerging challenge is not simply to perform fewer tests but to identify who truly benefits from additional testing and to select the right test for the right patient at the right time.

## 1. Introduction

Kidney transplantation (KT) remains the preferred treatment for eligible patients with end-stage kidney disease, offering substantial survival and quality-of-life benefits compared with maintenance dialysis [1]. Cardiovascular (CV) disease, however, remains a major determinant of morbidity and mortality before and after KT. Advanced chronic kidney disease (CKD) is characterized by a complex CV phenotype in which obstructive coronary artery disease (CAD) coexists with left ventricular hypertrophy, myocardial fibrosis, microvascular dysfunction, heart failure, arrhythmias, valvular disease, and accelerated vascular aging [2]. Identifying candidates at increased CV risk is therefore essential to KT evaluation.

For decades, this objective has largely been pursued through increasingly intensive screening for obstructive CAD, often with routine noninvasive stress testing followed by coronary angiography and revascularization when abnormalities are detected. Yet the evidence supporting this paradigm is remarkably limited. Screening practices vary widely, conventional tests imperfectly predict perioperative and long-term events, and no convincing evidence shows that systematic detection and prophylactic treatment of stable CAD improve outcomes in asymptomatic KT candidates [3]. Moreover, testing itself may have consequences: invasive procedures, revascularization, dual antiplatelet therapy, temporary wait-list inactivation, and delayed transplantation may offset uncertain CV benefits. These trade-offs are particularly relevant in KT, where clinical decisions must balance individual CV risk against access to a time-sensitive, potentially life-prolonging treatment [4].

Contemporary guidance increasingly recognizes these limitations. The 2022 American Heart Association Scientific Statement questioned the routine coronary screening of carefully selected low-risk candidates [3]. The recent British Transplantation Society/British Cardiovascular Society Guideline [5] explicitly encourages centers with historically high rates of noninvasive and invasive CV testing to critically review their protocols. The 2026 European Society of Cardiology Guidelines (ESC) on cardiovascular disease and CKD, developed in collaboration with the European Renal Association (ERA), further reinforce this direction. These guidelines propose a simplified risk-based approach and recommend against diagnostic CAD testing for KT candidates with a low probability of obstructive disease [6].

Yet these recommendations also reveal an important implementation gap. Evidence supporting CV assessment before KT remains limited, so many recommendations necessarily rely on low-level evidence. Major uncertainties persist about who should undergo additional testing, which tests should be preferred, how functional information should be interpreted, and how patients should be reassessed while awaiting transplantation. Consequently, contemporary guidelines define the direction of travel more clearly than the operational pathway, leaving substantial discretion to individual transplant centers. This is particularly relevant for historically high-testing centers seeking to move toward more selective assessment without compromising CV or transplant outcomes.

Against this background, the challenge is no longer simply whether CV testing before KT should become more selective but how to translate broad risk-based recommendations into clinically feasible pathways. In this viewpoint, we examine this implementation gap, discuss whether CV assessment should evolve from detecting obstructive CAD to a broader characterization of physiological vulnerability, and present one multidisciplinary pathway as an illustrative example of pragmatic protocol revision in a historically high-testing transplant center.

## 2. Why Is Protocol Revision Particularly Challenging in Kidney Transplantation?

One of the most underappreciated aspects of pre-transplant CV assessment is that transplantation medicine is guided by a transplant-specific mindset that may not be intuitive to many cardiologists. This may partly explain why CV evaluation before KT is often perceived as excessively intensive or overly conservative. Although KT is generally classified as an intermediate-risk noncardiac procedure [7], this classification does not fully capture its biological and organizational complexity. Perioperative CV events may compromise not only patient survival but also long-term graft function. Unlike most surgical complications, graft loss represents the irreversible loss of a scarce societal resource. Transplant physicians must therefore balance two partially competing principles: equity, ensuring access to transplantation for appropriate candidates, and utility, maximizing graft longevity and post-transplant outcomes [4]. This tension is particularly relevant in patients with substantial CV comorbidity, who may have both an urgent need for KT and an increased perioperative risk.

Kidney transplantation also differs fundamentally from conventional elective surgery because its timing is often unpredictable. Deceased-donor transplantation may occur within hours of organ availability, even after years on the waiting list, creating pressure to anticipate relevant CV problems before organ allocation. Conversely, living-donor transplantation is time-sensitive, and delays due to additional investigations, coronary intervention, or DAPT may jeopardize the transplant opportunity.

These challenges are particularly relevant after percutaneous coronary intervention (PCI). Because transplantation is often deferred until completion of DAPT and no randomized studies have established the optimal DAPT duration, stent selection, or alternative antiplatelet strategies for KT candidates [8], clinical decisions must weigh the uncertain long-term benefits of coronary revascularization against the tangible consequences of delaying transplantation.

Prolonged waiting itself may also be cardiotoxic. Progressive dialysis exposure is linked to left ventricular hypertrophy, myocardial fibrosis, vascular calcification, arrhythmias, and heart failure [9]. In patients with exhausted vascular access, recurrent dialysis-related infections, intradialytic instability, progressive frailty, or calciphylaxis, repeated invasive testing or prolonged DAPT may directly conflict with the urgency of transplantation. Thus, overly intensive CV assessment may paradoxically increase CV risk by delaying access to KT.

These considerations help explain why CV assessment before KT is inherently more complex than in most noncardiac surgical procedures. They also reinforce the need for strategies that are biologically meaningful, pragmatically feasible, and carefully balanced against the potential harms of overdiagnosis, overtreatment, and delayed transplantation.

Institutional performance metrics may also shape this historical approach. Transplant teams are accountable for perioperative events, post-transplant patient survival, and graft outcomes, and may understandably favor intensive preoperative investigation to minimize perceived preventable risk. Yet institutions should weigh these incentives against the consequences of testing cascades, temporary inactivation, and delayed access to transplantation. These competing pressures explain why a selective strategy requires transparent multidisciplinary agreement and prospective evaluation, rather than merely reducing the number of investigations.

## 3. The Cardiovascular Biology of End-Stage Renal Disease: Are We Screening the Wrong Disease?

The traditional paradigm of CAD screening assumes that CV events are primarily driven by flow-limiting epicardial coronary stenoses that ischemia testing can detect and that revascularization can modify. Although this model may remain applicable in earlier stages of CKD, it becomes progressively less representative as renal dysfunction advances. End-stage renal disease is characterized by a distinct CV phenotype that includes accelerated atherosclerosis, medial vascular calcification, chronic inflammation, endothelial dysfunction, left ventricular hypertrophy, myocardial fibrosis, autonomic dysregulation, microvascular dysfunction, and dialysis-related myocardial injury. Consequently, the spectrum of CV events shifts from predominantly atherosclerotic complications toward sudden death, arrhythmias, heart failure, and other non-atherosclerotic mechanisms [2].

This changing biology helps explain several apparent paradoxes in contemporary CV screening. First, diagnostic performance of functional stress testing remains highly variable among KT candidates. The most frequently cited systematic review, the 2011 Cochrane analysis, included only about 1300 patients across 25 heterogeneous studies [10]. Dobutamine stress echocardiography showed only moderate diagnostic accuracy, whereas myocardial perfusion scintigraphy performed even less consistently. The available evidence is further limited by small sample sizes, verification bias, heterogeneous populations, and the absence of contemporary modalities such as coronary computed tomography angiography, cardiac magnetic resonance, and cardiopulmonary exercise testing.

Second, inducible ischemia does not necessarily indicate obstructive epicardial CAD. In the International Study of Comparative Health Effectiveness With Medical and Invasive Approaches–Chronic Kidney Disease (ISCHEMIA-CKD) study, approximately one-quarter of patients with moderate-to-severe ischemia had no obstructive CAD on invasive angiography [11], underscoring the roles of microvascular dysfunction, impaired coronary flow reserve, and demand–supply mismatch. Consistent with this concept, an initial invasive strategy neither reduced death nor myocardial infarction [11] nor improved health status compared with conservative management [12].

These findings raise a provocative yet unavoidable question: are we screening for the wrong disease at the wrong stage of CKD? If advanced CKD is increasingly characterized by systemic myocardial and vascular vulnerability rather than by obstructive epicardial CAD alone, the objectives of CV assessment and the protocols designed to achieve them may also need to evolve.

ISCHEMIA-CKD compared initial invasive and conservative management in patients with advanced kidney disease and moderate-to-severe ischemia, not coronary screening versus no screening in asymptomatic transplant candidates. Importantly, a post hoc analysis of 194 participants listed for kidney transplantation found no significant reduction in death or nonfatal myocardial infarction with an initial invasive strategy (adjusted HR 0.91, 95% CI 0.54–1.54) [13]. Although these findings cannot establish the safety of omitting screening, they challenge the assumption that ischemia detection should routinely lead to invasive angiography or prophylactic revascularization.

## 4. When the Evidence Changes, Protocols Should Change Too

The intensity of CV screening before KT stands in stark contrast to the limited quality of the supporting evidence. Uncertainty extends beyond the diagnostic performance of individual tests to whether screening-triggered interventions improve patient outcomes. This evidence gap is reflected in the remarkable variability in clinical practice: US Renal Data System data showed that stress testing rates ranged from approximately 11% to 96% across transplant centers [14], suggesting that institutional philosophy may influence practice as much as patient-level risk.

More importantly, current evidence has not consistently shown that routine screening of asymptomatic KT candidates reduces perioperative myocardial infarction or early post-transplant mortality [15,16]. Although functional testing may provide midterm prognostic information [10], it remains uncertain whether this information leads to interventions that improve clinical outcomes.

The key practical question is no longer whether centers can perform more testing but whether centers with historically intensive screening protocols should reconsider their strategies. The contemporary challenge may instead be to identify candidates for whom routine ischemia testing can be safely omitted, thereby limiting downstream invasive procedures, DAPT exposure, and delays in transplantation. The 2022 AHA Scientific Statement already supported deferring routine stress testing in carefully selected very-low-risk candidates [3]. Current guidance has since made this direction more explicit.

The BTS/BCS Guideline [5] explicitly invites centers with high rates of noninvasive and invasive testing to review their protocols and audit outcomes. Its recommendations discourage routine further testing in asymptomatic patients with good exercise tolerance (Grade 2C) and leave the choice of functional or anatomical testing to local expertise and availability (Grade 2C). The ESC/ERA Guidelines [6] provide a risk-based screening algorithm and recommend against diagnostic CAD screening in candidates with a low probability of obstructive CAD due to potential screening-related harms, including delayed listing (Class III, Level C). Thus, selective testing is guideline-concordant care, not an untested relaxation of clinical vigilance. In candidates with relevant symptoms, established disease, or other guideline-defined indications, appropriate cardiovascular evaluation remains essential.

## 5. When More Testing Does Not Necessarily Lead to Better Outcomes

The management of stable CAD before KT remains supported by limited transplant-specific evidence. This uncertainty extends beyond screening to the downstream management of patients with CAD. A meta-analysis of 945 KT candidates with documented CAD found no reduction in all-cause mortality, CV mortality, or major adverse CV events with pre-transplant coronary revascularization compared with optimal medical therapy alone [17]. This exemplifies the prognostic–therapeutic gap in KT cardiology. Decisions about PCI, stent selection, and DAPT duration are largely extrapolated from the general cardiology literature, despite the substantially higher risks of restenosis, stent thrombosis, bleeding, and procedural complications in patients with end-stage renal disease [2]. Moreover, CV testing is not neutral: additional investigations may lead to invasive angiography, PCI, prolonged DAPT, temporary wait-list inactivation, and delays in transplantation.

The 2026 ESC/ERA Guidelines [6] reinforce this uncertainty, noting the lack of adequately powered contemporary randomized trials demonstrating benefit from CAD screening and recommending against invasive coronary angiography as first-line diagnostic testing for asymptomatic KT candidates. Current evidence therefore challenges the assumption that identifying more CAD necessarily improves patient outcomes and supports reconsidering screening pathways that routinely trigger downstream interventions without clear evidence of benefit.

## 6. Rethinking Functional Testing: From Ischemia Detection to Physiological Vulnerability

Perhaps the most important conceptual shift emerging from recent evidence is not the development of new stress tests but a reinterpretation of the information they provide. Traditionally, stress testing has been viewed primarily as a tool for detecting flow-limiting coronary stenosis. However, exercise performance may capture broader dimensions of CV vulnerability beyond inducible ischemia. A recent subanalysis of the ISCHEMIA trial illustrated this concept, including 1864 patients with moderate-to-severe ischemia and anatomically confirmed CAD on coronary computed tomography angiography [18]. Each additional metabolic equivalent (MET) achieved was associated with about a 14% reduction in all-cause mortality and provided substantial prognostic information beyond coronary anatomy. Thus, even in patients with significant ischemia and obstructive CAD, prognosis appears to depend not only on coronary stenosis but also on physiological reserve.

This perspective may be particularly relevant to KT candidates. In advanced CKD, impaired exercise capacity reflects the combined effects of frailty, sarcopenia, autonomic dysfunction, chronic inflammation, anemia, heart failure, pulmonary limitation, and microvascular disease. The treadmill test may therefore be viewed less as a pure ischemia detector and more as an integrated physiological stress test. Exercise stress echocardiography (ESE) is feasible in many KT candidates [19] and assesses ischemia, functional capacity, chronotropic competence, hemodynamic adaptation, symptoms, and ventricular performance. Observational data further show that each additional MET achieved during ESE is associated with a progressive reduction in major CV events, without an obvious threshold [20].

Exercise stress echocardiography can be particularly informative when patients are able to exercise, even if reduced exercise tolerance is anticipated. The achieved workload, symptoms, chronotropic and blood-pressure responses, and ventricular performance provide prognostic information beyond ischemia detection; a low workload may remain informative even when the exercise level is insufficient for a conclusive ischemia assessment. When patients cannot exercise, pharmacological stress imaging is an appropriate alternative if ischemia testing is clinically indicated. However, inability to exercise may itself identify patients at higher baseline risk: in a large observational comparison, those undergoing dobutamine rather than exercise stress echocardiography had less favorable survival, including after a negative stress study, although selection bias and confounding limit causal interpretation [21]. This does not establish diagnostic superiority of exercise stress echocardiography, demonstrate that acting on exercise findings improves transplant outcomes, or justify coronary screening or angiography solely because a patient cannot exercise.

This interpretation aligns with the 2026 ESC/ERA Guidelines [6], which recognize that functional testing in CKD may be more prognostic than diagnostic. For centers reconsidering their assessment pathways, exercise-based testing may help characterize physiological reserve and identify candidates who could benefit from prehabilitation, optimization of medical therapy, or closer longitudinal follow-up. Ultimately, if CV vulnerability in advanced CKD reflects systemic physiological impairment rather than isolated epicardial CAD, stress testing itself may not need to change; what may need to change is the clinical question we ask of it.

## 7. Revisiting Wait-List Surveillance: From Routine Retesting to Risk-Adapted Follow-Up

The optimal strategy for CV surveillance during active wait-list follow-up remains a major unresolved question in KT cardiology. Although repeated stress testing every 1–2 years is widely practiced, high-quality evidence supporting this approach is limited. Observational studies and cost-effectiveness analyses suggest that routine retesting may worsen outcomes by triggering unnecessary invasive procedures, delaying transplantation, and increasing exposure to contrast agents and DAPT [22]. Likewise, repeat coronary angiography in asymptomatic patients with known CAD lacks prospective evidence. The widely adopted “1–2-year rule” therefore reflects pragmatic practice rather than evidence-based medicine, and no randomized trial has compared scheduled retesting with clinically triggered reassessment [3].

The 2026 ESC/ERA Guidelines [6] reinforce a more selective approach: periodic CAD re-screening may be considered for wait-listed candidates with symptoms, prior abnormalities, or unexpected new cardiac findings, while acknowledging the uncertainty surrounding routine surveillance. For centers reviewing their protocols, this supports a shift from time-driven routine ischemia testing to risk-driven CV reassessment. Importantly, less routine CAD screening does not mean less CV care: follow-up frequency and intensity should remain individualized based on baseline risk, clinical evolution, functional capacity, and anticipated waiting time.

Annual echocardiography for patients with established structural heart disease and biennial echocardiography for those without known cardiopathy are pragmatic local surveillance intervals, not schedules established by randomized trials or formal recommendations for routine CAD re-screening. New symptoms, reduced dialysis tolerance, worsening functional capacity, suspected fluid overload, or new examination findings should prompt earlier clinical reassessment. Less routine ischemia testing must not translate into less active management of blood pressure, volume status, dyslipidemia, diabetes, heart failure, valvular disease, or modifiable frailty.

## 8. Translating Emerging Evidence into Clinical Practice: An Illustrative Protocol Revision

**Figure 1:**
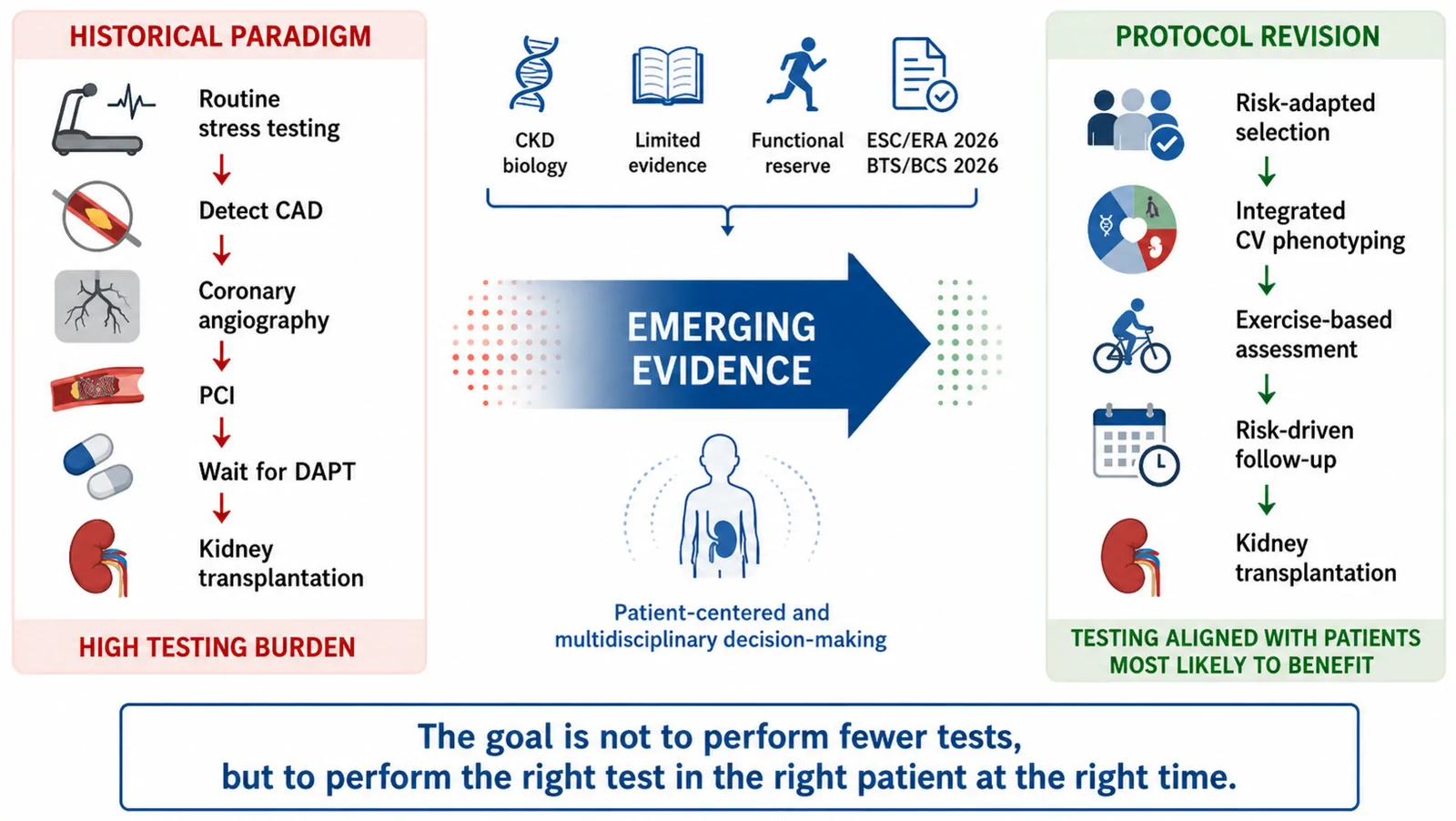
Revisiting cardiovascular assessment before kidney transplantation: translating emerging evidence into revised protocols. Contemporary evidence and guidance increasingly support a shift from routine, high-intensity cardiovascular testing to individualized, risk-adapted assessment. The illustration shows how these broad principles can translate into a clinically practical, physiology-oriented pathway through patient-centered, multidisciplinary decision-making.

**Figure 2:**
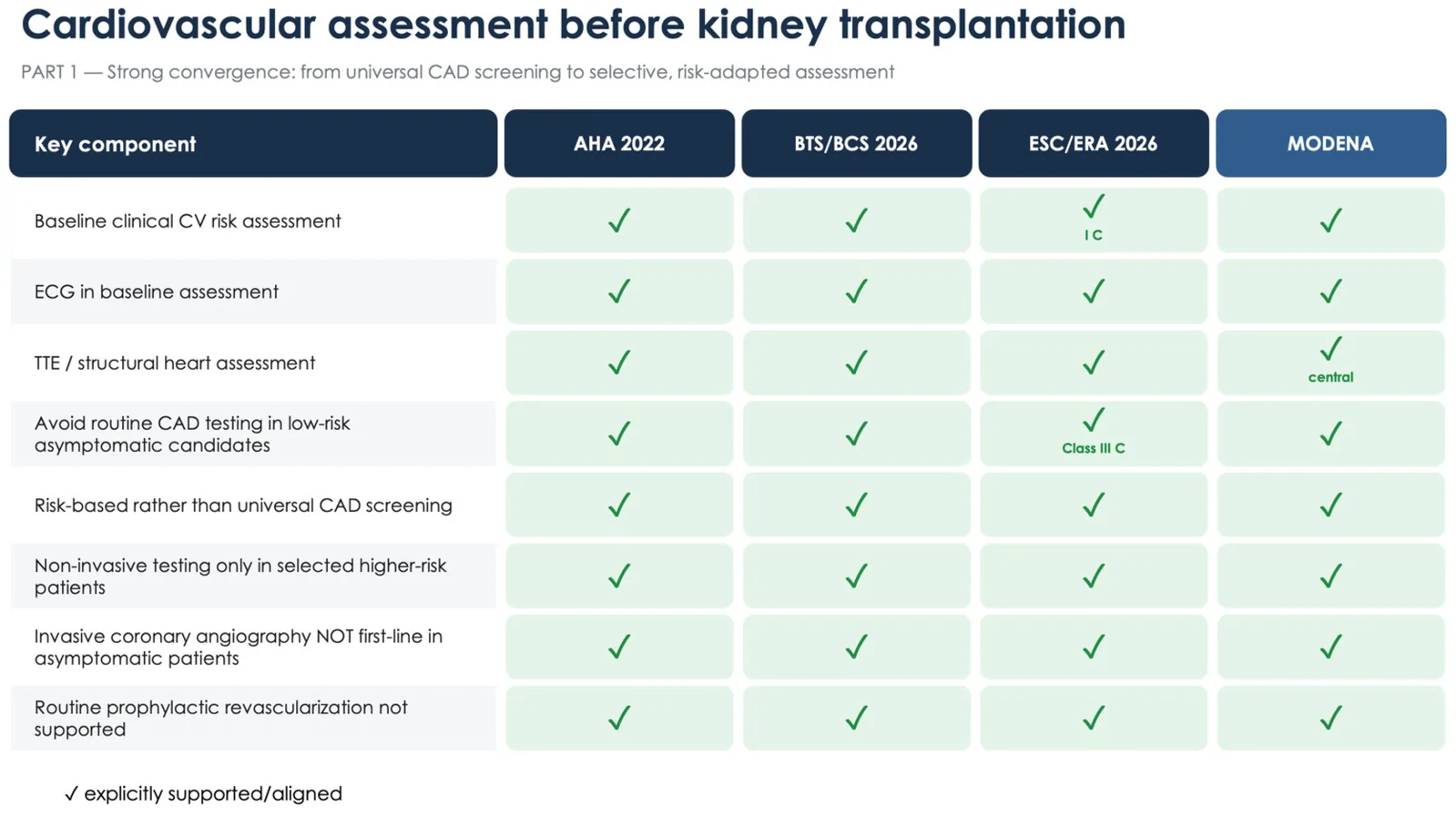
Guideline convergence in cardiovascular assessment before kidney transplantation. Qualitative comparison of selected principles from the 2022 AHA Scientific Statement, the 2026 BTS/BCS guideline, and the 2026 ESC/ERA guideline, alongside local implementation in Modena. Symbols indicate broad conceptual agreement, not equivalence in recommendation strength. The BTS/BCS guideline uses GRADE strength/evidence notation; the ESC/ERA guideline uses recommendation classes and evidence levels (see ESC/ERA Recommendation Table 32 [6]). The AHA 2022 document is a scientific statement. Formal examples: ESC/ERA baseline ECG and transthoracic echocardiography, I C; avoidance of CAD screening in candidates with low probability of obstructive disease, III C; BTS/BCS avoidance of routine further testing in asymptomatic candidates with good exercise tolerance, 2C. The ESC/ERA remains the reference algorithm for patient selection. CAD, coronary artery disease; ECG, electrocardiogram; TTE, transthoracic echocardiography.

**Figure 3:**
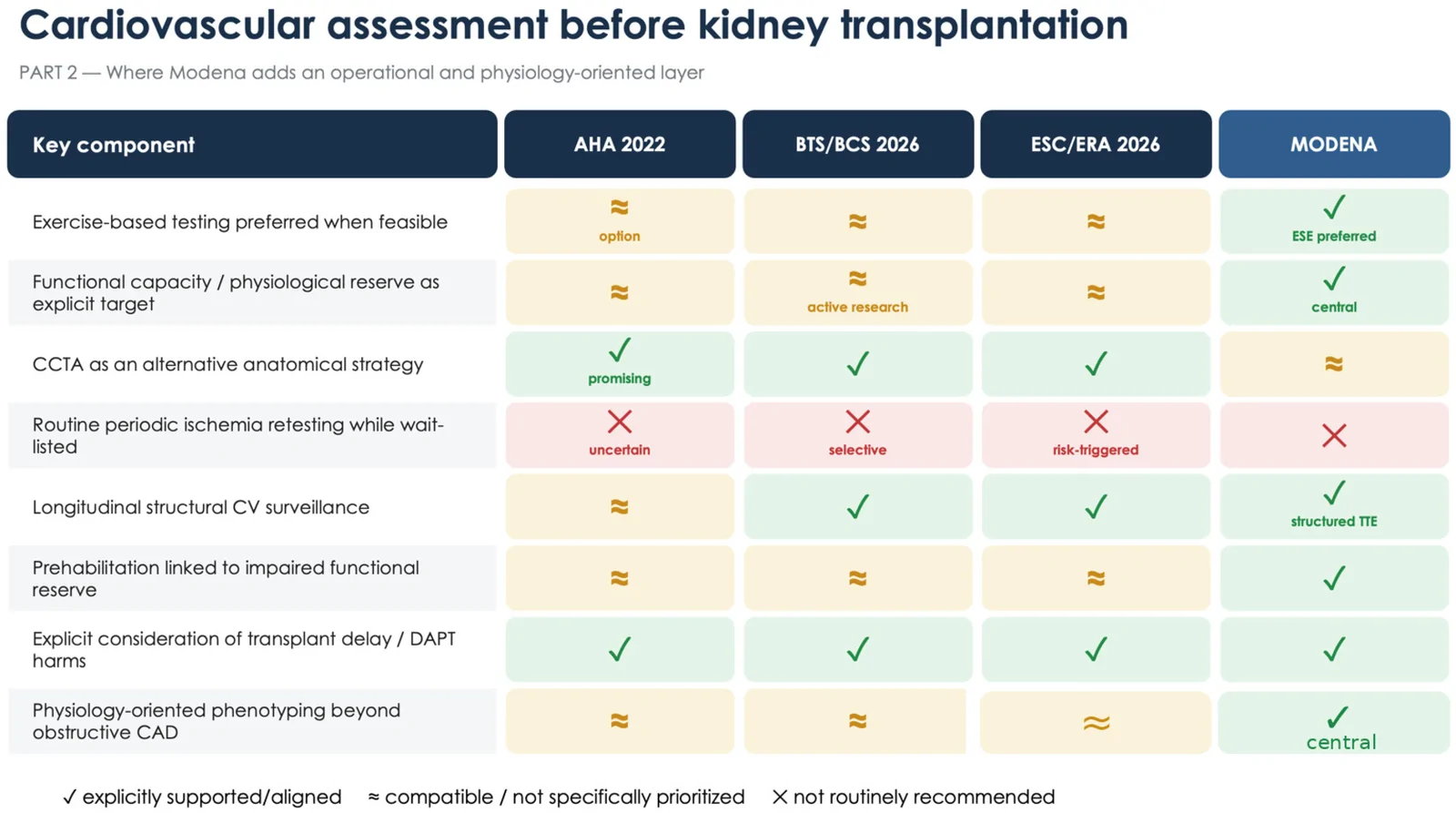
From guideline-directed patient selection to local test selection: the Modena experience. Qualitative comparison highlighting operational choices made by the Modena center, particularly a preference for exercise stress echocardiography when a functional test is indicated and the patient can exercise. This preference is not a specific ESC/ERA or BTS/BCS recommendation and does not imply diagnostic superiority over pharmacological stress imaging or coronary CT angiography. BTS/BCS supports choosing between functional and anatomical testing based on local expertise and availability (Grade 2C); the ESC/ERA guideline specifies its own modality recommendations for high-risk asymptomatic candidates. The figure does not replace the ESC/ERA screening algorithm. ESE, exercise stress echocardiography; CCTA, coronary CT angiography; DAPT, dual antiplatelet therapy.

The recently implemented Modena multidisciplinary pathway demonstrates how to apply the selective-testing principles of the 2022 AHA Scientific Statement [3] and the BTS/BCS guideline [5] in practice; it also aligns with the subsequently published ESC/ERA guideline [6]. When functional testing is indicated, ESE is preferred for patients who can exercise, including those with anticipated limited exercise tolerance, because it provides information on functional reserve in addition to inducible ischemia. Other stress-imaging or anatomical modalities remain appropriate based on the clinical question and local expertise. Table 1 and Figure 1, Figure 2 and Figure 3 summarize the evolving evidence and its local implementation.

The table summarizes the key evidence-based principles and contemporary guidance that informed the revision of the Modena cardiovascular assessment pathway for kidney transplantation. These principles reflect the evolving evidence base and recommendations from the 2022 AHA Scientific Statement and the 2026 BTS/BCS Guideline. The subsequently published 2026 ESC/ERA Guidelines further support several elements of this risk-adapted approach. The final column illustrates how these principles were applied in local clinical practice at a transplant center with historically high rates of noninvasive and invasive cardiovascular testing. The Modena pathway is presented as an illustrative example of knowledge translation rather than as a guideline recommendation, a validated alternative strategy, or a universally applicable model.

Developed through multidisciplinary collaboration between transplant nephrologists and cardiologists, the pathway shifts from a predominantly CAD-centered strategy to an integrated CV phenotyping approach. Echocardiography assumes a broader longitudinal role, assessing ventricular function, pulmonary pressures, valvular disease, regional wall-motion abnormalities, and structural CV vulnerability. When feasible, ESE is preferred because exercise performance provides prognostic information beyond ischemia detection.

During active wait-list follow-up, the pathway shifts from routine repeated ischemia testing to longitudinal, risk-adapted CV evaluation. Echocardiographic reassessment is performed annually in patients with established structural heart disease and every two years in those without known cardiopathy. These intervals are intended for surveillance of structural heart disease rather than routine CAD re-screening. Although randomized evidence does not support them, they reflect a pragmatic response to the dynamic CV phenotype of dialysis populations.

Routine ischemia testing is thus limited to selected patients, whereas structured cardiology follow-up addresses heart failure, valvular disease, arrhythmias, CV therapy optimization, and transplant-specific considerations, including DAPT-related delays and time-sensitive clinical trajectories. Whether this protocol revision safely reduces the testing burden without compromising CV or transplant outcomes remains unknown. Likewise, the historical high-testing strategy has never been prospectively validated against these outcomes.

Structural and hemodynamic assessment deserves equal attention. Diastolic dysfunction, elevated filling pressures, pulmonary hypertension, right ventricular dysfunction, and valvular disease are common in advanced CKD and are influenced by volume status. Echocardiographic findings should therefore be interpreted in the context of dialysis timing, blood pressure, and estimated euvolemia, with reassessment after volume optimization when appropriate [9]. Persistent pulmonary hypertension or unexplained right ventricular dysfunction warrants specialist evaluation and, when indicated, right heart catheterization to distinguish pre- from post-capillary disease. Exercise testing, including cardiopulmonary exercise testing, may complement resting imaging but cannot replace invasive hemodynamic assessment when necessary. These findings should guide dialysis optimization, medical management, and transplant planning.

The Modena pathway has only recently been implemented, so longitudinal outcomes cannot yet be meaningfully compared with historical practice. Its purpose here is to illustrate the local application of the BTS/BCS recommendation that centers with high testing rates critically reassess their protocols [5], not to claim superior clinical outcomes. Prospective assessment of CV events, downstream testing, waiting-list outcomes, and time to transplantation will be necessary.

## 9. Future Directions: From Better Tests to Better Decisions

Future research should shift from developing increasingly accurate diagnostic tests to determining who truly benefits from CV testing, how surveillance should be individualized, and which interventions meaningfully improve outcomes before KT. Exercise capacity and cardiopulmonary fitness may become increasingly relevant markers of physiological vulnerability, with cardiopulmonary exercise testing providing a more comprehensive assessment of functional reserve and frailty [23]. Artificial intelligence and multimodal predictive models may further refine individualized risk stratification by integrating anatomical, functional, biochemical, and clinical data [24].

Future strategies should also focus on modifying CV risk rather than merely identifying it, using structured prehabilitation, optimization of cardiorenal therapies, frailty-directed interventions, and personalized surveillance pathways [25]. Consistent with the 2026 ESC/ERA Guidelines [6], adequately powered prospective studies comparing alternative screening and surveillance strategies remain a major unmet need. The Canadian-Australasian Randomized Trial of Screening Kidney Transplant Candidates for Coronary Artery Disease (CARSK) [26], which compares ongoing routine CAD surveillance with no routine CAD surveillance after wait-listing, should provide important evidence to inform this question.

### What Evidence Is Required to Support a New Standard for Cardiovascular Assessment?

For a risk-adapted, physiology-oriented strategy to become standard practice, prospective comparative evidence is needed. The key question is not whether it reduces testing but whether it can do so without compromising CV or transplant outcomes. Ideally, pragmatic randomized or cluster-randomized studies should compare intensive screening with risk-adapted assessment and evaluate clinical outcomes, including downstream angiography, revascularization, DAPT exposure, wait-list inactivation, time to transplantation, healthcare costs, and false-positive cascades. External validation of patient-selection criteria and surveillance strategies will also be essential before broader implementation.

An equally important question is whether this level of evidence will ever be attainable. The biological complexity of advanced CKD, the heterogeneity of transplant candidates, and the practical challenges of conducting adequately powered randomized trials may make definitive evidence elusive. If so, the goal should be neither to preserve historical protocols by default nor to wait for perfect evidence but to continuously reassess clinical practice as evidence, pathophysiological understanding, and transplant experience evolve.

## 10. Conclusions

Cardiovascular assessment before KT is at a turning point. If more intensive CAD screening does not necessarily translate into better outcomes, the question is no longer how much we can test but who truly benefits from testing and which cardiovascular vulnerability we aim to detect. The future may depend less on performing more tests than on asking the right clinical question and selecting the right test for the right patient at the right time.

## Figures and Tables

**Table 1:** From evolving evidence to pragmatic protocol revision in cardiovascular assessment before kidney transplantation.

Key Domain	Evidence and Guidance Informing Protocol Revision	Illustrative Protocol Adaptation
Overall philosophy	From routine CAD screening toward proportionate, risk-adapted CV assessment that avoids unnecessary testing and delays to transplantation.	Critical review of a historically high-testing strategy.
Patient selection	Further CAD testing should be targeted to candidates most likely to benefit and avoided in those with a low probability of obstructive CAD.	Routine ischemia testing avoided in predefined low-risk groups.
Primary clinical objective	Beyond obstructive CAD detection toward broader characterization of CV risk and vulnerability.	Greater emphasis on structural heart disease, physiological vulnerability, and functional reserve.
Role of stress testing	Functional testing should be selectively used and may provide prognostic information beyond ischemia detection.	ESE preferred whenever feasible to integrate ischemia assessment with exercise capacity and physiological response.
Downstream management	Routine prophylactic revascularization is not supported by current evidence; invasive angiography should not be used as first-line testing in asymptomatic candidates.	Coronary angiography and PCI restricted to guideline-based clinical indications.
Wait-list surveillance	From routine scheduled CAD retesting toward clinically triggered, risk-adapted reassessment.	Risk-driven longitudinal CV follow-up rather than automatic periodic ischemia testing.
Transplant-specific perspective	CV decisions should account for potential harms of testing, transplant delays, and competing risks.	DAPT, donor availability, waiting time, and time-sensitive clinical trajectories incorporated into multidisciplinary decision-making.
Implications for high-testing centers	Contemporary guidance increasingly supports more selective testing; BTS/BCS specifically encourages high-testing centers to critically review their protocols.	One illustrative example of pragmatic protocol revision in response to evolving evidence.
Generalizability	Evidence supports principles rather than a single universally applicable pathway.	An example of knowledge translation rather than a validated or universally applicable protocol.

Abbreviations: BCS, British Cardiovascular Society; BTS, British Transplantation Society; CAD, coronary artery disease; CV, cardiovascular; DAPT, dual antiplatelet therapy; ESE, exercise stress echocardiography; PCI, percutaneous coronary intervention.

## Data Availability

No new data were created or analyzed in this study. Data sharing is not applicable to this article.
